# Supplementary material for: Learning curve and surgical outcome of robotic assisted colorectal surgery with ERAS program
Source: Sci Rep. 2022 Nov 29;12:20566. doi: 10.1038/s41598-022-24665-w (PMC9709162; doi:10.1038/s41598-022-24665-w)
Supplement: Supplementary file 2 — Supplementary Information 2. [file 41598_2022_24665_MOESM2_ESM.zip › RAL Raw data-Ñ[▒K/070/OP N_ñw╝╨░O▒Kñσ.pdf]

W92 / W92

臺中榮民總醫院
手術室護理記錄

[redacted]

基本資料

- 室別： 開刀房-17室，手術科別： CRS，手術類別： 常規，日期： 2021/04/01
- 進入時間： 1545 ； 麻醉開始時間： 1555 ； Time Out時間： 1750 ； 劃刀時間： 1752 ； 手術結束時間： 2210 送出時間： 2220 ；
- 麻醉方式： GE
  診斷：CA OF RECTUM
  手術名稱：Robot Assisted Low Anterior Resection
  手術局部用藥：NIL
  術前預防性抗生素：
  - 抗生素名稱一： Veterin 1GM IVD由麻醉護理師給予
  - 時間： 1730
  - 上次簽章: 李郁萱
  ，追加預防性抗生素：
- 手術醫師： 蔣鋒帆，楊曙榮，林陳彥，蔡易儒，林易萱
- 麻醉醫師： VS 陳永銘
-
  - 刷手護士A ： 廖子晴，時間： 1545，刷手護士B ： 何佳青，時間： 1545，接替刷手一： 廖子晴，時間： 1730
  - 流動護士A ： 李郁萱，時間： 1545，接替流動一： 廖子晴，時間： 1630，接替流動二： 李郁萱，時間： 1700
- 更多接替及流動護士： 接替流動三： 盧陵怡，時間： 2000，接替流動四： 周筱涵，時間： 2030，接替流動五： 林書綾，時間： 2130

皮膚狀況

A-V Shunt： 無
術前： 完整
記錄者： 上次簽章: 李郁萱
術後： 完整
記錄者： 林書綾

手術準備

手術臥式 單一手術臥式

-
  - 手術臥式： 垂頭仰臥式
  - 使用附件： 溫毯 38 ℃，頭圈，腳架，側身架，其它： SILICON PAD
  - 皮膚準備： 10%Aq Betadine，Hibiscrub，2% Chlorhexidine
  - 電燒： 使用電燒: 電極片位置： 右大腿，記錄者： 上次簽章: 李郁萱

止血帶T： 無

植入物

- 植入物名稱/號碼/數量/部位：DAVINCI XI STAPLER 45mm x2/rectum

，記錄者： 林書綾

標本送檢

- 細菌培養 無
- 細胞學 無
- 冰凍切片 無
- 病理標本
  組織名稱一：IMA ROOT
  ，
  組織名稱二：DISTAL CUT END
  ，

組織名稱三： RECTUM
- 截肢 無

標本交付 標本： 1 袋 ， 標本： 0 盒 ， 標本： 2 瓶 ， 細胞學： 0 管 ， Culture Tube： 0 管 ， 被交付醫師： R 林易萱，
- 記錄者： 林書綾
- 時間： 2210

傷口術後敷料

傷口敷料： 傷口敷料： 無
- 請於圖上標示部位：

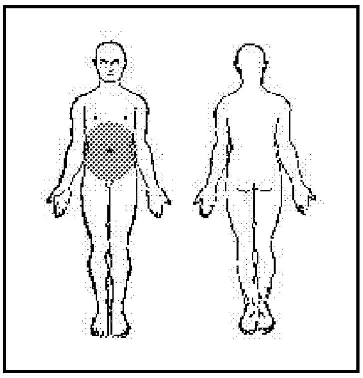

- 傷口敷料： MEPORE

導管

導管	天數	導管資訊	插入日期	插入時間	更換日期	拔除日期	拔除時間	拔除原因	預計拔除/更換	預計拔除/更換日期
Foley(1)	1	號碼:16 Fr.; 部位:膀胱;	2021/04/01	1630						
A-line (1)	1	號碼:20 G; 部位:左手;	2021/04/01	1625						
Double Lumen CVC(1)	1	號碼:8 Fr. Fr.; 深度:15cm; 部位:jugular (右); 象限:RU;	2021/04/01	1620						

用物計數

- 手術中出血量： minimal c.c.
- 手術中尿量： 450 c.c.
-
  - 其它： 無

用物計數： 多切口同時縫合
- 1.器械： 正確，2.縫針： 正確，3.刀片 ： 正確，4.紗布： 正確，5.腹部墊： 未使用，7.PEANUTS ： 未使用，8.紗條： 未使用，9.絲帶： 未使用，10.針頭： 未使用，11.Vessel loop： 未使用，12.Boots： 未使用，13.Jelco： 未使用，14.棉條： 未使用，
  - 第一次：
    - 刷手護士 ： 廖子晴
    - 流動護士： 上次簽章: 周筱涵
    - 時間： 2120

  ,
  - 第二次：
    - 刷手護士 ： 廖子晴
    - 流動護士： 林書綾
    - 時間： 2135

  ,
  - 第三次：
    - 刷手護士 ： 廖子晴
    - 流動護士： 林書綾
    - 時間： 2145

備註

備註： OLYMPUS SCOPE NO.1
DAVINCI SCOPE 30度 NO.1

- 記錄者： 上次簽章: 李郁萱
- 時間： 2000

填寫護理人員 林書綾

正式摘要

W92 / W92

doctorRank:B
